# Role of Conserved Non-Coding Regulatory Elements in LMW Glutenin Gene Expression

**DOI:** 10.1371/journal.pone.0029501

**Published:** 2011-12-29

**Authors:** Angéla Juhász, Szabolcs Makai, Endre Sebestyén, László Tamás, Ervin Balázs

**Affiliations:** 1 Applied Genomics Department, Agricultural Research Institute of the Hungarian Academy of Sciences, Martonvásár, Hungary; 2 Department of Plant Physiology and Molecular Plant Biology, Eötvös Loránd University, Budapest, Hungary; Louisiana State University and A & M College, United States of America

## Abstract

Transcriptional regulation of LMW glutenin genes were investigated *in-silico*, using publicly available gene sequences and expression data. Genes were grouped into different LMW glutenin types and their promoter profiles were determined using *cis*-acting regulatory elements databases and published results. The various *cis*-acting elements belong to some conserved non-coding regulatory regions (CREs) and might act in two different ways. There are elements, such as GCN4 motifs found in the long endosperm box that could serve as key factors in tissue-specific expression. Some other elements, such as the AACA/TA motifs or the individual prolamin box variants, might modulate the level of expression. Based on the promoter sequences and expression characteristic LMW glutenin genes might be transcribed following two different mechanisms. Most of the s- and i-type genes show a continuously increasing expression pattern. The m-type genes, however, demonstrate normal distribution in their expression profiles. Differences observed in their expression could be related to the differences found in their promoter sequences. Polymorphisms in the number and combination of *cis*-acting elements in their promoter regions can be of crucial importance in the diverse levels of production of single LMW glutenin gene types.

## Introduction

Wheat is among the three mayor crops in the world used for human consumption and animal feeding. Cereals serve as a major source in calories and proteins in human nutrition. Prolamins, the storage proteins of wheat play a crucial role in the quality of flour based end-products. Their allelic structure and expression largely determine the strength and extensibility of dough made from wheat flour. Prolamins consisting of monomeric gliadins and polymeric high molecular weight (HMW-) and low molecular weight glutenin subunits (LMW-GSs) form a complex protein matrix, called the gluten. The composition and size of this gluten highly depends on the expression profile of the different components.

The LMW glutenin subunit gene family is encoded at the Glu-3 loci on the short arm of homeologous chromosomes 1A, 1B and 1D, respectively. Based on their N-terminal sequences they can be grouped into three main groups. These three main groups were initially characterized by their mature peptide sequences starting with either isoleucine (LMW-i-type), serine (LMW-s-type) or methionine (LMW-m-type) at their N-terminal [1], [2], [3]. Although i-type genes are exclusively expressed in the A genome of wheat, most types are expressed at multiple genomes parallel. If the number and position of cysteine residues is also considered, 12 distinct groups can be characterized [4]. However, following the grouping system of Ikeda the homogeneity found in some of the LMW groups was too low, compared to other groups. If sequences belonging to the same Ikeda group were aligned some groups could be further clustered [5]. After a thorough characterisation of the LMW glutenin sequences some specific short polypeptide motifs positioned either close to the N-terminal region or mostly in the C-terminal region have been identified. Using these characteristics several further LMW glutenin gene types, as well as some sub-types could be distinguished (Table 1). (Alignment of deduced amino acid sequences of each LMW glutenin gene types is presented in Supplemental material File S1). Storage protein genes are specifically transcribed in developing wheat seeds, and their expression is controlled primarily at the transcriptional level [6], [7]. The synthesis and deposition of seed storage proteins (SSPs) is subject to strict spatial and temporal regulation, which occurs specifically only in the endosperm during the seed development. Their expression sensitively depends on the availability of nitrogen and sulphur in the grain [8] and shows similar profiles, indicating a remarkable conservation in the machinery responsible for the maturation gene expression programmes. Common regulatory elements, such as *cis*-acting elements and *trans*-acting transcription factors observed in monocot SSP genes have already been reviewed [9], [10], [11]. The −300 element or endosperm box is conserved at around 300 nucleotides upstream of the transcriptional start site in most of the cereal storage protein families [12], [13], [14]. The endosperm box is approximately 30 nucleotides long and is considered to be primarily responsible for the endosperm-specific expression. It consists of two *cis*-elements, the prolamin box (P-box) and the GCN4-motif [15]. The GCN4 motif, also known as the nitrogen element (N motif) was originally found to be similar to the GCN4-binding motif in yeast [16]. Altogether seven motifs have been identified in storage protein gene promoters in *Poaceae* [10]. Based on these results most of the *Poaceae* SSP gene promoters are characterized by one GCN4 motif, two P-box, one Skn-1 and one GCAA motifs. The Skn-1 like motif is a half site of the palindrome sequence recognized by the Storage Protein Activator transcription factor (SPA) and was originally identified in *C. elegans*. It also has been identified in storage protein gene promoters of other cereals [10], [17], [18]. The GCAA motif is a highly conserved DNA sequence in the promoters of storage protein genes found in rice [19] and maize [20], thereby implying the possibility of their controlling role in transcription. Next to these elements some other motifs, such as the motif weakly similar to the maize Opaque 2 (O2) bZIP recognition site have been described. Alongside these elements, the AACA/TA motif, ACGT motif and CCAAT motif was also suggested as being involved in the prolamin gene expression regulation [15], [19].

**Table 1:** Classification of the LMW glutenin genes types according to different grouping methods.

LMW glutenin gene type^a^	N-terminal	LMW main type^b^	IkedaType/Group^c^	Locus	>300	Reference accession
IQA	ISQQQQAPPFS	LMW-i	VI/11	Glu-A3	−	X07747
IPP	ISQQQQPPPFS	LMW-i	VI/11	Glu-A3	+	FJ447464
IQP	ISQQQQQPPFS	LMW-i	VI/12	Glu-A3	−	FJ876821
IEN	IENSHIPGLEK	LMW-s	II/4	Glu-B3/Glu-D3	+	AY542898
MEN	MENSHIPGLEK	LMW-s	-	Glu-B3 ?	+	AY608420
MSP1	MENSHIPGLER	LMW-s	II/3	Glu-B3	+	FJ447463
MSP2	MENSHIPGLER				−	EU369715
MSP3	MENSHIPGLER				−	EU369722
MSG1	METSHIPGLEK	LMW-m	I/1	Glu-D3	+	EU189096
MSG2	METSHIPGLEK				−	EU369734
MSH	METSHIPSLEK	LMW-m	I/2	Glu-B3/Glu-D3	+	EU189089
MRC1	METRCIPGLER	LMW-m	V/10	Glu-D3	+	EU189094
MRC2	METRCIPGLER				+	FJ615311
MDS1	MDTSYIPGLER	LMW-m	IV/6	Glu-A3	−	FJ549936
MDS2	MDTSCIPGLER				−	FJ549935
MSC1	METSCIPGLER	LMW-m	IV/8	Glu-A3/Glu-D3	+	EU189091
MSC2	METSCIPGLER		IV/9		−	AY994358
MSS1	METSCISGLER	LMW-m	IV/7	Glu-D3	−	AY831795
MSS2	METSCISGLER				+	DQ457416
MSV	METSRVPGLEK	LMW-m	III/5	Glu-D3	+	EU189098

Grouping method a is used in the present study ; method b is reviewed by D'Ovidio and Masci 2004 [65]; method c is based on Ikeda et al., 2002 [4]. The present study uses a classification method which is based on the thorough comparison of the entire coding region [5]. Sequences longer than 300 nucleotides were used for the promoter analyses, and they are labelled with a +sign. Shorter sequences or where no sequence information was available for LMW glutenin gene types were labelled with a - sign.

Based on our current knowledge there are three main transcription factor families involved in the endosperm-specific transactivation of the prolamin genes, the bZIP, the DOF and the MYB transcription factors. Basic leucine zipper (bZIP) TFs have broad binding specificity, binding not only to the ACGT core sequences, but also to the GCN4 core (TGAGTCA) in wheat prolamins or to the O2 recognition site in maize (TCCACGTAGA) [21]. Active function of bZIP transcription factors is highly dependent on N availability. These transcription factors are stably expressed upon amino acid starvation, and degrade as the amino acid level reaches the demanded level in the cells [22]. The Prolamin-box is recognized by plant-specific DOF transcription factors that contain one zinc-finger domain. The wheat prolamin box binding factor (WPBF) *trans*-activates the storage protein genes via binding to the AAAG core motif in their promoter sequence [23]. Plant MYB transcription factors represent a large family of proteins which include the conserved MYB DNA-binding domain in one, two or three copies. Its members, the R2R3-MYB factors, are involved among others in regulation processes related to flowering and seed development [24]. The AACA/TA motif is the core binding site of HvGAMYB, OsMYB5 and TaMYB transcription factors in barley, rice and wheat respectively [25], [26], [27].

Transcriptional regulation and the number and composition of *cis*-acting elements show some variation in the different prolamin gene families. Sulphur rich prolamins (LMW glutenins, gamma- and alpha-gliadins), as well as sulphur poor omega-gliadins, possess similar regulatory system. The TATA box sequence CTATAAA is highly conserved in all the prolamin genes and is positioned around −60 nucleotides upstream of the start codon. The presence of the bipartitate endosperm box at the position −300 is also characteristic of most of the prolamin gene families, except for the promoters of HMW glutenins. Some prolamin genes contain additional complete or partial endosperm boxes. The number and position of these additional elements shows some differences among the gene families. Shewry and co-workers have reviewed these endosperm boxes and based on their opinion the additional complete or partial binding sites do not seem to be required for the initial promoter activity and are not present in gamma or omega-gliadin promoters [9]. Basic studies related to the transcriptional regulation of LMW glutenin genes have been published after the isolation of the LMW glutenin *LMWG1D1* gene [28]. Two prolamin boxes, a CCAAT-motif and a TATA-box were identified in the first 300 nucleotides upstream of the start codon [28]. Based on similarities to other cereal storage protein promoters the −300 element containing both the prolamin box and the GCN4-like motif has also been identified with an additional copy of each motif in close vicinity [15]. These two copies of endosperm boxes form a so-called long endosperm box (LEB) [29]. These studies were focusing on the identification of *cis*-acting elements as well as basic processes through which the *LMWG1D1* gene is regulated. Papers related to the promoter analysis of the *LMWD1D1* gene describe the mechanisms of transcriptional regulation only of one of the LMW glutenin gene types. To better understand the roles of different LMW glutenin types in the machinery of gluten formation, it is necessary to begin by assessing the promoter characteristics of this gene family. In this study, the 5′ non-coding regions of most of the LMW glutenin gene types were characterized. Expressional control of LMW glutenin genes has been analysed *in-silico*. Current knowledge on *cis*-acting regulatory sequences and possible interacting transcription factors attached to these LMW glutenin promoters were modelled.

## Materials and Methods

### Data collection and sequence characterization

Plant genomic sequence databases have been screened for LMW glutenin genes. More than 360 LMW glutenin sequences have been identified, from which genes with promoter sequences have been collected and used for further analyses. Altogether 170 LMW glutenin genes have been identified with promoter lengths between 30 and 2008 nucleotides (for sequences see Supplemental material File S1). Only promoter sequences over 100 nucleotides in length were used for further analyses. These sequences included only the 5′ non-coding regions of LMW glutenin genes from *Triticum aestivum* L. and most of the i-type, m-type and s-type sequences identified previously were represented (Table 1) [5], [30]. For details of sequence alignment of characterised LMW glutenin gene types see Supplemental material File S2. Non-coding *cis*-acting elements have been identified using published results and plant promoter databases like PLACE or PlantCARE [31], [32]. Promoter profiles were graphically represented using the Promoter Profiler tool developed by one of the authors. The presence or absence of the various *cis*-acting elements was indicated by a 1 or 0 for each accession analysed and was collected into a data matrix. Accessions belonging to the same LMW glutenin gene type were aligned and compared using the tools DIALIGN [33] and CLC Genomic Workbench (CLC Bio). Consensus promoter sequences were generated using the algorithm of CLC Genomic Workbench and were used to characterise the promoter types. Conserved regulatory element regions (CREs) were identified manually after sequences were aligned.

### Method for calculating gene expression based on EST libraries

Due to the high sequential similarity of LMW glutenin genes, we did not use clustered ESTs for the apparent error rates of clustering [34]. A BLAST search with strict parameters was used instead, and all hits were analysed and judged individually. We ran the search with the LMW genes as queries, and used the EST sequences of the selected developing seed libraries representing 80567 ESTs as the search set (the *blastn* parameters were as follows: *-word_size: 128, -ungapped, -mismatch_penalty: −30*). In the next step, high scoring segment pairs (HSPs) were filtered to count hits only with 100% identity. All other results were discarded.

Several queries had the same EST hits, as a result of high sequence homology between LMW genes, so they had to be compared and classified individually, as a given EST can only belong to one query sequence. Each EST was assigned to the gene where it showed the longest aligned length and highest hit identity.

Publicly available EST data from developing wheat seed libraries of Glenlea (NCBI EST library identifiers #A9H, #A9I and #A9J), Chinese Spring (#10469, #19547, #10479, and Mercia (#FIN, #FIQ, #FIT) cultivars and a genotype of unknown origin published by DuPont (#BSD, #BSE, #BSF, #BSH, #BSG) have been used for the analyses. All expression data were in a timeframe of 3 to 30 days after flowering. Expression data were normalized against total EST hits found in the single libraries and described as Transcript Per Million EST values (TPM).

### Statistical analyses

Cluster analysis was used to group promoters with similar profiles. LMW glutenin sequences were characterized based on the *cis*-acting elements observed in their promoter regions. The presence or absence of promoter motifs was denoted with a 1 or 0 in a data matrix. These data were used for K-means clustering using the Statistica 8.0 (StatSoft Inc.) program package. Prior to the analysis, the number of clusters were determined using the tree joining clustering algorithm. The two-way joining clustering method was used to describe relationships between LMW glutenin gene types and promoter motif patterns. The aim of this analysis was to discover promoter motifs or promoter profiles directly related to the individual LMW gene types.

To be able to compare the abundance of transcripts belonging to the same gene-, promoter-types or CRE groups in the different EST libraries a log-likelihood ratio statistic was carried out using the method of Schaaf et al (2005) [35]. The log likelihood ratio test (G-test) offers the null hypothesis (all libraries have the same EST proportion), as well as a straightforward way to proceed when the null hypothesis is rejected, and the EST proportions in the libraries are not equal. The latter approach can be adapted to fit the design of a particular expression analysis using subsets of cDNA libraries. The G-test procedure assumes the sampling or within-library variation to be binomially distributed. The use of the G-test on contingency tables of libraries and specific and non-specific EST counts can be envisioned as an extension of the use of the Chi-squared test for the comparison of two libraries. Expression analyses comparing levels of transcripts among the different libraries have been carried out. Results were statistically confirmed using the G-test and represented using the tool of Pavlidis and Noble (2003) [36].

The function of CREs in gene expression was investigated using the same statistical methods as used above. Accessions possessing the same CRE patterns were clustered together. *Cis*-acting elements outside the CRE regions were excluded from the analysis. Expression levels of different CRE groups during the seed development were charted and significant differences were calculated using the G tests.

## Results

**Figure 1:**
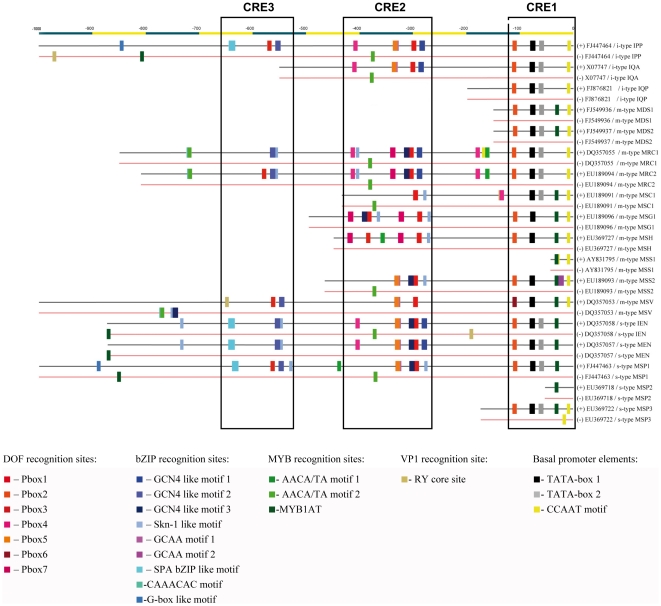
Promoter regions of the different LMW glutenin gene types. The first 1000 nucleotide region upstream of the transcriptional start of LMW glutenin gene types is presented through specific examples. Accessions of genes possessing the presented promoter regions are indicated. Both sense (+) and anti-sense (−) strands are presented. Conserved non-coding regulatory element regions are labelled with rectangles CRE1, CRE2 and CRE3. The identified cis-acting elements are labelled with different colours.

Based on published results and plant promoter databases 24 variants of 12 different SSP gene specific *cis*-acting promoter motifs have been identified (Table 2). Three different variants of a GCN4 like motif and seven different variants of prolamin boxes have been identified in the LMW glutenin gene promoters. All the P-box motifs were identified based on sequence identities found in wheat or other cereal species. P-box7 (TGTAAAAGT) was originally identified in pea legumins [37]. LMW glutenin gene type promoters (Figure 1) were described using sequences chosen after comparing the promoters of each of the types separately. Polymorphism identified in the different endosperm boxes, including the presence or absence of a complete LEB is presented in table 3. Promoter profiles of individual gene types are presented in the following section and are discussed based on their main types, i-, s- and m-type, respectively. Most of the *cis*-acting elements identified in the promoter sequences of LMW glutenin gene family are concentrated in the first 1000 nucleotides upstream of the start codon.

**Table 2:** *Cis*-acting elements identified in the promoters of LMW glutenin gene types.

Name	DNA binding motif	Transcription factor	References
(CA)n element	CAAACAC	bZIP	Stalberg et al., 1996 [66]
CCAAT box	CCAAT	CBF	Albani and Robert 1995 [67]
G box like element	TGACGT	bZIP	Menkens et al., 1995 [68]
GCAA motif1	GCAAAAGTG	bZIP	Takaiwa et al 1996 [19]
GCAA motif2	GCAAAAGTA	bZIP	present study
GCN4 like motif 1	GTGAGTCAT	O2 bZIP	Albani et al., 1997 [29]
GCN4 like motif 2	ATGAGTCAT	O2 bZIP	Müller and Knudsen 1993 [16]
GCN4 like motif 3	GTGTGACAT	O2 bZIP	Albani et al., 1997 [29]
AACA/TA motif 1	TAACAA	R2R3 MYB	Diaz et al., 2002 [25]
AACA/TA motif 2	AACAAA	R2R3 MYB	Diaz et al., 2002 [25]
MYB1AT core	AAACCA	R2R3 MYB	Abe et al., 1997 [69]
P-box 1	TGTAAAGT	PBF DOF	Kreis et al., 1985 [70]
P-box 2	TGCAAAG	PBF DOF	Sugiyama et al., 1985 [71]
P-box 3	TGTAAAG	PBF DOF	Colot et al., 1987 [28]
P-box4	TGCAAAC	PBF DOF	Norre et al., 2002 [72]
P-box5	TGCAAAAG	PBF DOF	Norre et al., 2002 [72]
P-box6	TGGAAAG	PBF DOF	US Patent Application 20090064374 [76]
P-box7	TGTAAAAGT	PBF DOF	Shrisat et al., 1989 [37]
RY core site	CATGCA	ABI3/VP1	Suzuki et al., 1997 [73]
Skn-1 like motif	GTCAT	bZIP	Blackwell et al., 1994 [74]
SPA bZIP	GATGACGTGTC	O2 bZIP	Mena et al., 1998 [38]
TATA-box 1	CTATAAATA	TBP	Bernard et al., 2010 [75]
TATA-box 2	GTATAAAG	TBP	Bernard et al., 2010 [75]
TATA like motif	TATAA	TBP	Bernard et al., 2010 [75]

DNA binding motif – recognition sites, Transcription factors – bZIP- basic leuzin zipper, CBF – CCAAT binding factor, O2-bZIP – TF similar to Opaque 2 transcription factor, R2R3 MYB – MYB transcription factor with two or three binding motif, PBF DOF – prolamin box binding factors of the DNA-binding with one finger domain transcription factor family, ABI3/VP1 – Abscisic acid insensitive3 or viviparous1 transcription factors, TBP - TATA binding protein. Papers reporting the elements in wheat or other cereal storage protein gene promoters are presented.

**Table 3:** Polymorphism observed in the endosperm boxes of LMW glutenin gene types.

LMW glutenin type	LEB	LEBcomposition		additional EB
		−300 element (EB1)	EB2	
IQA	−	Pbox1 – GCN4 like 1	Pbox5	no data
IPP	−	Pbox1 – GCN4 like 1	Pbox5	Pbox3 - GCN4 like 3
IQP	−	Pbox1 – GCN4 like 1	Pbox5	no data
IEN	+	Pbox1 – GCN4 like 1	Pbox5 – GCN4 like 3	–
MEN	+	Pbox1 – GCN4 like 1	Pbox5 – GCN4 like 3	–
MSP1	−	Pbox1 – Skn1 like	Pbox 5- GCN4 like 3	Pbox3 – GCN4 like 2-Skn1 like
MSP2		no data	no data	no data
MSP3		no data	no data	no data
MSG1	−	Pbox1 – Skn1 like	Pbox7	Pbox 7 –GCN4 like 3
MSG2	no data			Pbox7 – Skn1 like
				no data
MSH	−	Pbox1 – Skn1 like	Pbox7	
MRC1	+	Pbox 1- GCN4 like 1	Pbox 7- GCN4 like 3	Pbox 4 – Skn1 like, Pbox3- GCN4 like 2-Skn1 like
MRC2	+	Pbox 1- GCN4 like 1	Pbox7- GCN4 like 3	
MDS1	no data	no data	no data	no data
MDS2	no data	no data	no data	no data
MSC1	−	Pbox1 – Skn1 like	–	no data
MSC2	no data	no data	no data	no data
MSS1	no data	no data	no data	no data
MSS2	−	Pbox1 – Skn1 like	Pbox5- GCN4 like 3	no data
MSV	−	Pbox1	Pbox5	Pbox3- GCN4 like 2

EB1 – endosperm box at the position −300 is the first EB in the Long Endosperm Box, EB2 – second endosperm box in the LEB; +- gene type contains a complete LEB, − gene type does not contain a complete LEB; no data – no sequence information is available.

### LMW i-type genes

LMW-i type gene promoters cannot be characterized by the presence of a LEB; however, they contain an additional EB around at the −550 position. Next to these two complete EBs, there are more P-boxes in the promoters at positions −112, −337 and −411. A bZIP recognition site, SPA bZIP has been identified around at the position −650 upstream of the start codon. The MYB1AT element is missing in the promoter of LMW-i type genes, except in some genes with an N-terminal of ISQQQQQQ(Q)PF, which tends to be an inactive form, due to the inner stop codons in the coding gene sequences. The i-type genes starting with ISQQQQPPPFS contain a RY core motif at the position −139, close to the double TATA box. All of the promoter sequences contain an AACA/TA motif 2 on the antisense strand around at the position of −380. RY core motifs have been identified on the antisense strand between the positions of −877 and −974. Additional MYB core motifs (AACA/TA motif 1 and 2) were identified further upstream in the sequences, at the positions-1037 and -1666.

### LMW s-type genes

Mature LMW glutenins starting with a serine belong either to the MSP, IEN or MEN gene types. MSP genes are exclusively encoded at the Glu-B3 locus and can be categorised at least into three different groups based on their sequence characteristics. Their promoter sequences, annotated as MSP1, MSP2 and MSP3 in this paper, also show some differences. In hexaploid wheat, only MSP1 type gene promoters were found with a length of over 300 nucleotides and some MSP3 type promoters were identified with a shorter length. The only difference we could identify among the MSP type promoters was that these promoters do not possess the CCAAT motif at the position −10 upstream of the start codon, except for MSP3 type gene promoters. MSP type genes do not have a complete LEB at position −300, however, LEB is characteristic of both MEN and IEN type genes. MSP1 genes possess an additional complete EB, located at the position of −560 accompanied by two or three Skn-1 like elements, and an AACA/TA motif at −441. Two additional MYB core motifs have been identified in the distal promoter region, at −1788 and −1794. Similar to the i-type sequences the MSP1 type genes also have a SPA bZIP motif around at the position −640. G-box like elements have been also identified at the positions of −891 and −1760, in proximity to one of the MYB recognition sites. All MSP promoter sequences contain the MYB1AT core element at −34.

The IEN and MEN type LMW glutenins are located at the Glu-D3 locus and show similar characteristics in their encoding genes. The promoter regions of IEN type genes show some common characteristics with the promoter region of MSP type genes, like the presence of SPA bZIP at the position −640. Further P-boxes have been found at positions −112 and −406. A single GCN4 like motif followed by a double Skn-1 like element was identified at −557 upstream of the start codon. A Skn-1 like motif was found further upstream in the sequence at −735 both in MEN and IEN type genes. This latter group also possesses an ACGT motif at −193.

The antisense strands of s-type genes also contained some conserved transcription factor recognition sites. All of the sequences analysed contain an AACA/TA motif 2 at −374, and additional MYB motifs (MYB1AT core) have been found in MSP1 and IEN gene promoters. Anti-sense strands also contained some RY core elements, conserved at the position of −193 in IEN type promoters and −1019 in MSP1 promoters.

### LMW m-type genes

LMW m-type sequences are the most diverse group containing at least seven different gene types. Based on the sequences of their encoding genes some of them can be further divided into subtypes (Table 1). MDS1 is a group of pseudo genes, containing inner stop codons. In some cases, like in groups of MDS types and MSG2, the promoter sequences found did not exceed 150 nucleotides in length. Even in these cases the elements positioned closest to the transcriptional start site (TATA box, CCAAT box and MYB1ATcore element) were conserved. LEB is not generally characteristic of m-type genes, except for MRC genes (Table 3).

METRCIPGLER type genes are one of the most abundant m-type groups, with two subtypes MRC1 and MRC2. Next to the LEB, their promoters contain an additional complete endosperm box at −582 nucleotides upstream of the start codon. Further P-boxes are found at the positions −416 and −182. These P-box motifs are not followed by GCN4 motifs, but are accompanied either by a Skn-1 like motif or an AACA/TA motif. A P-box2 motif is also found close to the double TATA box. No conserved MYB1AT core motif has been found in any of the promoters belonging to the MRC type genes.

MSV type gene promoters show an entirely different profile from the rest of the m-type genes. This group does not possess a complete −300 element. However, a complete endosperm box (P-box3 + GCN4 element 2) was found at the position- 565 upstream of the transcriptional start site. Similar to other m-type promoters a P-box6 motif is preceding the conserved TATA box. No double TATA box was identified in these gene promoters, but the MYB1AT element was present at the position −34. An additional RY core motif has been identified in the distal region (at −651) of these promoters.

Promoters of the MSH type LMW glutenin genes are present in two different forms, with several common motifs in their sequences. These promoters contain a MYB core element about 30 nucleotides upstream of the truncated LEB. Further conserved P-boxes were identified at the positions −112 (P-box2), −387 (P-box3) and −422 (P-box7), respectively. The MYB1AT motif is also present at −34. Sequences belonging to METSHIPGLEK type genes can be divided into two subgroups, MSG1 and MSG2, from which MSG2 type genes are pseudo genes. The most distinctive difference found between MSG1 and MSG2 gene promoters is that MSG2 promoters carry an extra MYB core element in proximity to the conserved MYB1AT core at position −34. MSG1 genes contain only a truncated element. An additional EB has been identified at −421 upstream of the transcriptional start site. A fifth prolamin box a P-box2 motif is present 32 nucleotides upstream of the TATA box. Sequences starting with METSCISGLER can be also divided into two subtypes. Based on our recent knowledge no promoter information is available for MSS1 type genes, except for some sequences originating from *Triticum tauschii*. Promoters of MSS2 genes possess an incomplete LEB. Similarly to other m-type gene promoters a P-box2 motif is positioned at −113, followed by a double TATA box1 and a TATA box like motif. MYB1AT core is also present at −34.

METSCIPGLER type LMW glutenin gene sequences can be also sub-grouped into two distinct groups, from which MSC1 sequences are more common in bread wheat. Some of the MSC2 genes contain inner stop codons. MSC1 type promoters do not have a LEB. Their −300 element is also partial, containing a P-box3 at −299 and a Skn-1 like element. A second prolamin box (P-box4) is positioned at −137 upstream of the start codon. The position of this P-box is slightly shifted compared to the P-boxes of the other m-type genes in the relevant regions and overlaps with a RY core site at −139. The antisense strands of m-type sequences also contain some additional elements. The m-type genes, with a cysteine at the 5^th^ position of their N terminal domain (MRC, MSC, MSS) all include an additional AACA/TA motif 2 at the position-375. This motif is in the neighbourhood of a P-box motif on the sense strand in most of these genes. This MYB core motif is absent in the rest of the m-type sequences. The MSV type promoter sequences include a small group of adjacent motifs at −772, comprising an AACA/TA motif 2 followed by a Skn-1 motif and a GCN4 like motif.

### Expression analysis of LMW glutenin EST sequences

**Figure 2:**
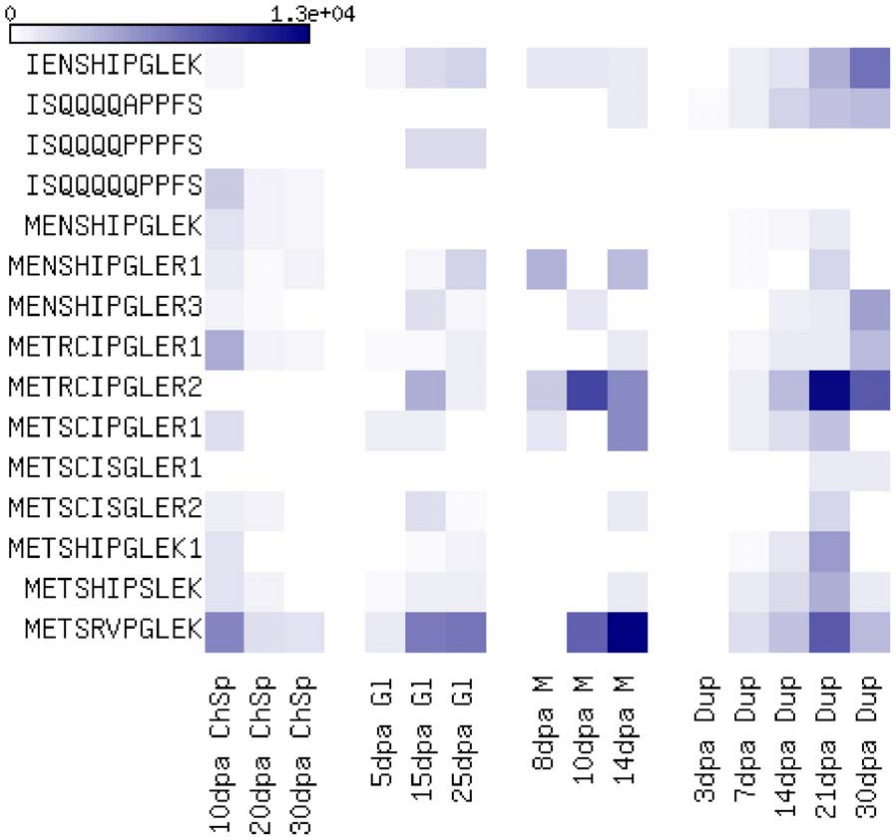
Comparison of expression profiles. ChSp, Gl, M and Dup abbreviations are for cDNA libraries originated from developing seeds of cultivars Chinese Spring, Glenlea, Mercia and an unknown genotype published by DuPont respectively. EST counts are labelled with colours, the smallest EST values are labelled with white, and the largest EST counts are signed in dark blue. 10 dpa, 20 dpa, etc. represents libraries isolated from seeds at 10, 20, etc. days after anthesis.

Expression levels of LMW glutenin gene types varied due to the differences observed in their allelic composition. Some gene types, like MSS1 or MEN were underrepresented. Significant differences were identified at p = 0.05 among MRC and MSV type genes in the Glenlea and Chinese Spring libraries (Figure 2). Tendencies observed for the rest of the gene types indicate that LMW glutenin gene expressions follow two different profiles. A group of genes (i-type genes and most of the s-type genes) show a continuously increasing level of transcription during development, while the other set of genes (e.g. m -type genes with cysteine in the fifth position) take a normal curve distribution in their expression profile. Their transcriptional maximum is reached at the mid-phase of the grain filling around at 25 DPA. Allelic effects resulted in significantly different expression profiles of the same LMW GS gene type, like in the case of MRC genes in Glenlea and Chinese Spring. In Glenlea, the MRC2 genes were strongly expressed, and the level of expression reached the maximum at around 21 DPA. In cv. Chinese Spring, the other gene type MRC1 was expressed, and MRC2 was present only in a negligible amount. For the detailed G test results see supplemental material File S4.

### Comparison of the promoter types

**Figure 3:**
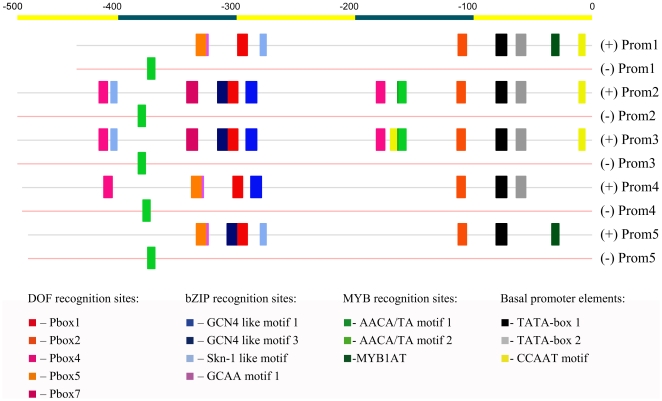
Promoter profiles of consensus promoter types. Prom1, Prom2, Prom3, Prom4 and Prom5 represent groups of accessions possessing the same promoter profiles. Clustering was made using the K-means clustering method and the promoter motif matrix as input data. Accessions belonging to the same promoter cluster were aligned and their consensus sequence was used to present characteristic cis-acting elements. Motifs present on both sense (+) and antisense (−) strands are represented with different colours.

A two-way joining cluster analysis was carried out to discover relationships between LMW glutenin gene types and their promoter profiles (Supplemental material File S3). There were motifs whose presence or absence was unique to some of the LMW glutenin gene types, like the presence of the MYB core motif at the position −164 in MRC type gene promoters or absence of the Skn1 like motif at the position −278 in MSV type promoters. However, using the available data set no significant relationship could be depicted. This suggests that there is a smaller level of polymorphism detected in the promoters compared to the coding sequences of the different LMW glutenin types. To reveal common characteristics in the promoters of the different gene types a comparative cluster –analysis was carried out using the promoter motif patterns of the analysed LMW glutenin gene accessions. The comparison was carried out on a sequence set containing *cis*-acting elements located in the 460 nucleotides long upstream region and resulted in five promoter types (Figure 3). Characterization of the different promoter groups has been carried out using the consensus sequences of each promoter group. Based on the consensus sequences only a few elements seemed to be conserved in all five groups. The position of the double TATA box and a P-box2, about 34 nucleotides further upstream of the TATA box1 was highly conserved. Four of the five groups possess a complete (Prom2 and Prom3) or partial LEB (Prom4, Prom5). Only two promoter types, Prom1 and Prom5 possessed a MYB1AT motif at the position −34. Prom2 and Prom3 promoter types possessed a MYB core motif (AACA/TA motif1) at the position −164. These two MYB core motifs were followed by a prolamin box (P-box4). Another P-box4 motif was found at −412 in promoter types 2, 3 and 4, respectively. Two of these P-box 4 motifs (in Prom2 and Prom3) are accompanied by Skn-1 motifs. Promoter types Prom2 and Prom3 are almost identical, except for a CCAAT motif identified in Prom3 type at −170.

Prom1 included sequences from the MSV, MSG, MSC and MSH type sequences. Prom2 and Prom3 consisted of MRC2 and MRC1 type sequences respectively. The MEN, IEN and i-type (IAP, IPP and IQP) promoters grouped into Prom4, while Prom5 consisted of MSP1 and MSS sequences.

**Figure 4:**
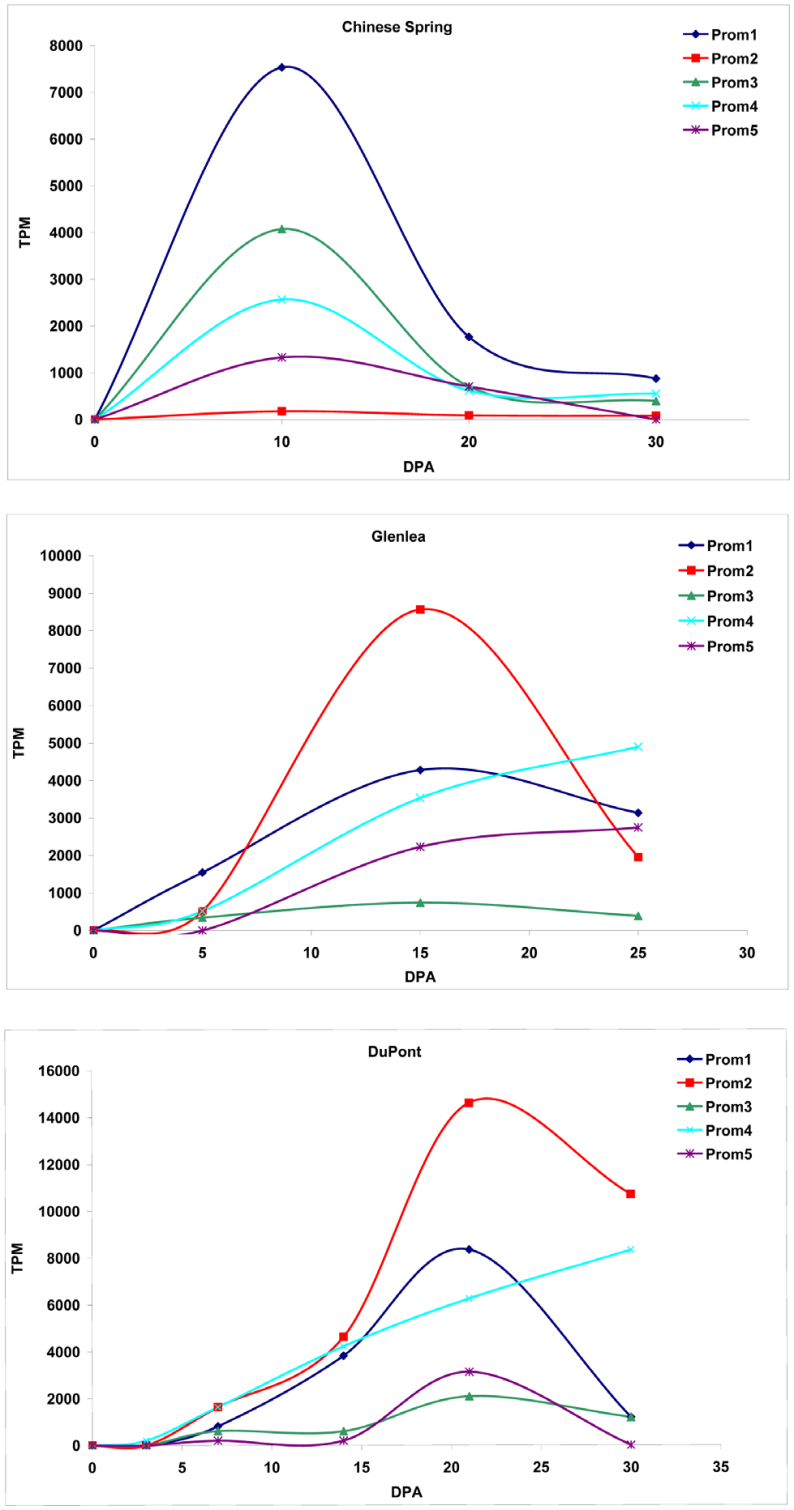
Expression profiles of genes with different promoter profiles. Expression profiles are presented for the libraries originating from the same genotype. TPM - normalised EST counts in transcript per million values. DPA – development stages in days after anthesis.

Using these datasets relationships between promoter profiles and expression profiles were tested. Based on the results of the log-likelihood ratio analysis all five promoter types showed significant changes in the levels of transcripts. For the detailed G test results see supplemental material File S5. Figure 4 presents expression profiles of LMW glutenin genes belonging to the same promoter types in cvs. Chinese Spring, Glenlea and the genotype by DuPont. As it is seen both in Glenlea and the unknown genotype, the expression levels of Prom2 type genes were the highest. However, these types did not express in a high amount in Chinese Spring, and Prom1 type genes showed the highest expression levels. Most of the promoter types followed a normal distribution in time, with a maximum level between 10 and 25 DPA. However, gene types with promoter profiles Prom4 did not reach their maximum in the mid-phase of the seed-development, which indicates a longer operability of the transcriptional apparatus.

LMW glutenin gene promoters were aligned and examined for regions possessing highly conserved *cis*-acting elements. Three conserved non-coding element regions were identified (Figure 1). The first conserved regulatory region (CRE1) contains motifs up to −120 nucleotides upstream of the transcriptional site. This set of *cis*-acting elements includes the TATA boxes, a CCAAT motif, a MYB1AT motif, a GCAA motif2, a CAACAC motif and a P-box2 motif. The second group of elements contains motifs between the −300 and −420 nucleotides, while the third CRE group contains promoter motifs between −500 and −650. CRE2 included elements of the long endosperm box surrounded by several Myb core motifs and some further elements. CRE3 included the region around at the position −550 containing another complete endosperm box, some Skn-1 like motifs and an SPA bZIP motif.

**Figure 5:**
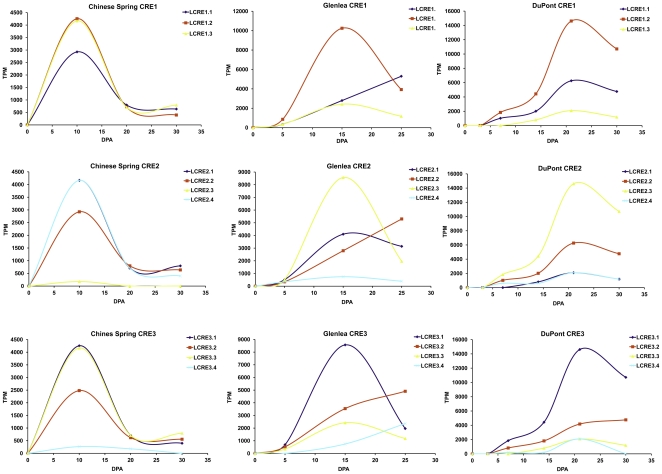
Effect of conserved noncoding regions on expression levels. Expression levels of accessions belonging to the same CRE type are presented in cultivars Glenlea, Chinese Spring and the unknown genotype by DuPont for all three CRE regions. CRE1.1, CRE2.1, CRE3.1 etc. represent groups of accessions possessing the same CRE pattern in the relevant CRE1, CRE2 or CRE3 region, respectively.

In CRE1 regions three distinct motif patterns were observed, while in both CRE2 and CRE3 regions there were four distinct motif clusters to be distinguished. Significant differences were calculated using the G tests. For the detailed results see Supplemental material File S6. Based on the comparative analysis, accessions with the same motif pattern in CRE1 followed a similar distribution during the seed development, not depending on the genotypes analysed (Figure 5). If motifs of CRE2 region were also considered, accessions with the same motif order did not share similar expression profiles in the different genotypes. However, if *cis*-acting elements in CRE3 were considered accessions belonging to the same CRE set showed similar expression profile in each genotype.

## Discussion

Based on the present knowledge there are no published results reviewing the unique expression and transcriptional control of the LMW glutenin gene family. To understand more detailed aspects of expression and control of these genes, 5′ non-coding regions of the publicly available LMW glutenin gene sequence have been analysed. Most of the elements we have found were already identified in cereal storage protein gene promoters, but were not discussed in detail in the case of the LMW glutenin genes. The promoter of the LMW glutenin gene *LMWG1D1* was previously used to describe basic rules of tissue-specific expression in wheat endosperm [15]. Primary *cis*-acting elements, such as the prolamin box and the GCN4-like motif at the position −300 and the transcription factors bound to them have been previously identified. In this study a more detailed analysis has been carried out, and variants of known elements have been described. We have identified some other elements which are present in proximity to each other and seem to be conserved in several gene types.

### Characterization of *cis*-acting elements in LMW glutenin gene promoters

In vivo footprinting and electrophoretic mobility shift assays revealed that the P-box within a LMW glutenin gene promoter is recognised by the WPBF, a member of the DOF transcription factors [38]. All DOF proteins analysed so far, except for a pumpkin protein [39], recognized an AAAG motif flanked by slightly different DNA sequences as the essential sequence element in DNA-binding assays in vitro [40], [41]. This indicates that DNA sequences bordering the AAAG core-motif have limited impact on the DOF–DNA interaction [42], although they might result in a reduced level of expression [43]. The prolamin box taken out of the context of its native promoter functioned as an active *cis*-acting element not only in cultured endosperm cells but also in leaf-tissue derived suspension cells [44].

Seven different P-box recognition motifs and three GCN4 like motif variants have been characterized in the present study. Both P-box and GCN4 variants are present in different number and position in the different gene type promoters. A duplicated endosperm box or long endosperm box might be characteristic of the LMW glutenin gene promoters, but was also found in some gamma gliadin gene promoters [45]. Our results indicate that the LEB motif is characteristic but not common in all members of the LMW glutenin gene family. It was present in gene types like IEN, MEN or MRC, while the rest of the LMW GS gene types contain only partial duplicates, missing either one or both of the GCN4-like motif elements. In this latter group of promoters, the P-boxes are usually followed by Skn-1 like motifs. Skn-1 transcription factors bind to the half sites of the recognition sites of GCN4 type bZIP TFs. It is quite possible that these two transcription factor types are competitors for the same recognition site. It is not known whether they induce the same changes, but they both seem to have a positive regulatory role [17].

In most of the gene types, complete endosperm boxes have also been found in other positions. The number of complete EBs and their position, especially their proximity to other transcription factor recognition sites might explain some of the differences observed in the transcriptional levels of these genes (Figure 2). In some cases, such as in MRC type genes, an additional complete endosperm box at about 300 nucleotides upstream of the LEB might have a primary role in the abundant transcription (Figure 1). However, the highest expression level, which was found in the group of MSV type genes, could not be related to the presence of an increased number of complete endosperm boxes. On the contrary, their −300 elements are not complete; the promoter sequences possess only two DOF binding sites (P-box3 and P-box5) in the LEB region without any bZIP TF binding site in their vicinity. Similar to the MRC type genes they do contain a complete EB in the −550 −580 region. To what extent this additional EB region is responsible for the differences observed in their expression level needs to be clarified with experimental data, even though it is much more likely that additional proximal *cis*-acting elements participate in the transcriptional regulation.

Such potential *cis*-acting elements, like the AACA/TA motifs or GCAA motifs might have an additional moderating effect on the transcriptional control of storage protein gene expression.

The GCAA motif is a highly conserved motif found in different positions in the promoters of cereal storage protein genes, and is a binding site for several bZIP TFs [19]. This motif overlaps with an A+T-rich region in rice, was also found in proximity to MYB1AT core and TATA boxes in one of the LMW glutenin types (MSS2). The importance of A+T-rich elements for quantitative expression has been demonstrated in various plant genes [46], [47], [48]. The AACA/TA motif was identified as one of the binding sites of MYB transcription factors in cereals [25], [26]. Several different types of MYB binding sites have been found in the different LMW glutenin gene promoters close to one of the DOF or bZIP TF recognition sites (Table 2). The AACA/TA motif 1 is in 20 nucleotides distance from P-box4 in both MRC type genes and was found at a 30 nucleotides distance in the MSH type genes. A more conserved AACA/TA motif 2 (at −375) was found on the anti-sense strand in about 60% of the gene types. This motif is located at 25 nucleotides distance from a DOF binding site (P-box4), except for gene types MSC1 and MSS2. These two gene types show a relatively low level of expression, which might be directly related to the absence of this motif. The same AACA/TA motif is also at 70 nucleotides proximity to the following GCN4+P-box motif pair of the LEB region in MRC, MSS, MSP, IEN and i-type genes. Another highly conserved MYB TF recognition site is present at 34 nucleotides upstream of the start codon. This motif, which was identified in the promoter of a dehydration-responsive gene in *Arabidopsis thaliana* might have a repressing role in the later phase of endosperm development. The MYB1AT motif is highly conserved in most of the LMW glutenin gene types, except for the MRC type gene promoters and some of the i-type promoters.

The CCAAT box is one of the most common elements in eukaryotic promoters. While many plant genes do not include a CCAAT motif in their proximal promoter, this seems to be another shared feature among seed storage protein genes [49]. Genes under the control of promoters containing CCAAT boxes can be tissue- and/or stage-specific suggesting that patterns of gene expression are also determined by other *cis*- and *trans*-acting factors. CCAAT boxes are highly conserved within homologous genes across species in terms of position, orientation, and flanking nucleotides [50]. They are typically found between 60–100 nucleotides upstream of the transcription start site and are operating in both direct and inverted orientations [51], [52], [53], [54]. However, in most of the LMW glutenin gene types CCAAT box motifs are located at 10 nucleotides upstream of the transcriptional start site.

It seems that only s-type genes (MSP, IEN, MEN) do not contain this motif. If we consider that transcript levels of these gene types do not follow a normal distribution during the grain filling, but seem to reach their expression maximum later, the absence of this element might have a stabilizing function in the transcriptional apparatus.

### Role of promoter profiles and conserved non-coding element regions

The composition and distribution of *cis*-acting elements in LMW glutenin gene promoters indicate that the position and order of some of the motifs might be more conserved compared to other motifs. Some of these conserved sequences are overall characteristic of storage protein gene promoters in cereals, such as the −300 element, while others might be gene family or chromosome specific.

If promoters based on their motif patterns were grouped, the expression analysis of their genes showed some statistically significant differences. Most of the genes followed a normal distribution in time, with a maximum level between 10 and 25 DPA. The other batch of genes did not reach their maximum transcript level in the mid-phase and seem to express for a longer time. Genes with promoter types Prom4 and Prom5 belong to this latter group, both consisting s-type and i-type LMW glutenin gene sequences mainly. The Prom1, Prom2 and Prom3 groups consist mainly genes with m-type sequences. As s-type sequences are mainly encoded at chromosome 1B and i-type sequences are expressed exclusively at chromosome 1A, this difference observed in their 5′ non-coding sequences might arise from the evolutionary differences of their genome progenitor species. In contrast with our expectations, there were some striking differences in the expression patterns of genes belonging to the same promoter types depending on the cultivar analysed. The most obvious difference was seen in the profiles of Prom2 and Prom3. Both promoter types belonged to MRC type genes. While in Chinese Spring MRC genes with Prom2 type promoters were expressed only in insignificant amounts, this promoter type seemed to be the most abundant both in cv. Glenlea and the unknown genotype by DuPont. An opposite trend was observed in the case of the expression of genes with Prom3 type promoters. Prom3 genes were expressed in high amounts in Chinese Spring but were found only in low levels in Glenlea and DuPont. Analysing the allelic composition of the cultivars Glenlea and Chinese Spring, as well as the genes belonging to the Prom2 and Prom3 types, it became clear, that the difference was rather allelic. Glenlea possess the *Glu-D3c* allele which consists mainly of m-type gene sequences, among them METRCIPGLER type sequences and on the other side Chinese Spring possess a *Glu-D3a* allele. One of the differences between *Glu-D3a* and *c* alleles is that while *Glu-D3a* contains mainly MRC1 type genes with Prom3 promoter profiles (e.g. FJ615311) *GluD3c* possess MRC2 type genes (e.g. EU189094) with Prom2 promoters.

Our results have shown that some gene types share similar promoter characteristics and possess similar conserved non-coding sequence regions. Conserved non-coding sequences were identified in cereals using comparative analysis of different grass genomes [55]. Cereal genomes exhibit a high level of synteny and sequence similarity within coding regions, facilitating the identification of orthologous gene sequences. Despite the relatively low overall sequence identity among promoter sequences from cereal genes compared with their coding regions, numerous expression analyses and transgenic studies involving promoter-target gene constructs have demonstrated that orthologous genes from different cereal species often share highly similar patterns of gene expression. Thus, these conserved patterns of gene expression might be programmed by regulatory sequences associated with conserved non-coding regions. This sequence conservation is also characteristic on the prolamins of *Triticeae* [56]. It has been reviewed by Shewry et al. (1994) that a large number of seed proteins have similar sequences to the proposed ancestral prolamin, which are also present within the unique regions of the *Triticeae* cereal prolamins [57]. The earliest divergence from this sequence was the insertion of repetitive sequences, to produce the LMW-GS protein and the HMW-GS protein groups of genes. This early step in prolamin evolution can be supported by the low level of significant similarities shared in these prolamin types' promoter sequences. The ancestral LMW-GS sequence diverged to give rise to the various sub-groups of sulphur-rich gliadins, on the ground of small insertion and deletion events across the gene, whereas the promoter regions remained relatively similar [56]. A conserved non-coding sequence survey of LMW glutenins might be a reasonable method to discover some intra-family characteristics.

As LMW glutenin gene promoters were examined, three conserved non-coding regulatory element regions (CREs) were identified. All three CREs contained several different *cis*-acting elements, which were conserved in all LMW glutenin gene types. However, there were elements whose sequences showed some polymorphisms. If the polymorphism found in the CRE patterns was related to expression profiles, it became obvious that CRE regions might have different regulatory roles in the transcription. Motif patterns in CRE1 and CRE3 resulted in similar expressional profiles independent from the genotypes analysed. *Cis*-acting elements in these regions are responsible for quantitative differences observed among the different LMW glutenin gene types. Polymorphism detected in the CRE2 region did not have an impact on the differences observed on transcription level in the genotypes during the seed filling. CRE2 harbours the LEB including the N-responsive GCN4-like motif in the −300 element. This region might play a key role in the tissue-specific regulation and might have less effect on quantitative differences observed in transcript abundance. The conserved presence and function of this region in other cereal and monocot species proves the existence of a less diverged storage protein promoter, compared to the polymorphisms detected in the coding sequences [56].

### Model for the transcriptional regulation of LMW glutenin gene expression

There are several models published to describe basic mechanisms and TF interactions involved in the transcription of cereal storage protein genes, including that of the LMW glutenin genes [11], [15], [29], [58]. All models emphasize the importance of the bipartitate endosperm box, containing a functional GCN4-like motif and a prolamin box. The bZIP and DOF transcription factors bound to these elements interact coordinated and play a crucial role in the regulation of tissue-specific seed-storage protein gene expression. The transcription factor bound to the GCN4 core element might require two or more partners to form a functional unit [43]. The model of Kawakatsu and Takaiwa (2010) demonstrates the binding of an Opaque2 TF homo-dimer to the transcription factor recognition sites in maize zein genes [11]. The effect of these trans-actions was modulated by the presence of MYB transcription factors, bound to AACA motifs. The AACA motif was able to suppress the expression of glutelin genes in vegetative tissues in transgenic rice experiments [59]. The MYB binding sites were found in the neighbourhood of both P-box and GCN4 like motifs of rice and barley genes [19], [25], [43], [60], [61]. MYB TFs seem to have a significant role at the beginning of storage protein expression which is underlined by their expression profiles compared to bZIP and DOF TFs [25], [62], [63]. Both MYB and DOF TFs have been expressed simultaneously at 10DPA in barley endosperm. Therefore, the pattern of HvGAMYB transcript accumulation was consistent with the possibility of HvGAMYB being a regulator of the Hor2 hordein gene [25]. Hammond and Kosack et al. (1993) described that at 13 days after flowering only P-box recognition sites were occupied in the −300 element of LMW glutenin promoters, but no GCN4 bZIP trans-activation has been observed at this stage [15]. Expression levels significantly increased from 18DPA after bZIP transcription factors were bound to their GCN4-like recognition motifs.

The use of different fragments of the *LMW1D1* promoter has proven the altered effect of several *cis*-acting elements in the tissue-specific expression [29]. A significant level of *uidA* gene expression in vegetative tissues of transgenic tobacco was observed when a promoter containing a prolamin box only, without a GCN4-like motif was used [29]. However, using different lengths of *LMW1D1* promoter fragments, including both GCN4-like and Pbox motifs no GUS expression could be detected in vegetative plant tissues [64]. A *LMW1D1* promoter variant containing the first 938 nucleotides resulted in significantly increased levels of expression compared to the shorter version of the same promoter. The longer promoter, which included an additional complete endosperm box showed a similar developmental onset and an increased level of GUS activity during seed development in transgenic wheat experiments [15], [64].

**Figure 6:**
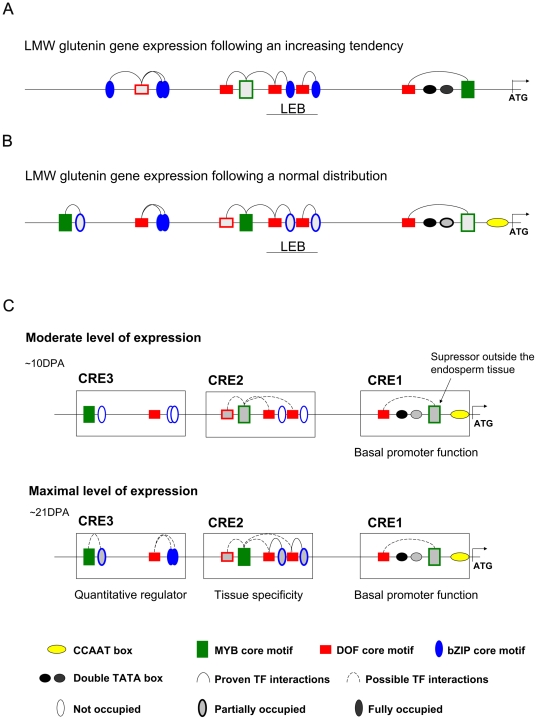
Model for the transcriptional regulation of LMW glutenin gene expression. Intensity of geometric forms marks the frequency of these elements present in the promoter types or developmental phases. Most important cis-acting elements and the possible TF interactions are labelled. A - Promoter profile of LMW glutenin genes following a continuously growing expression; B – Promoter profile of gene types following a normal distribution in expressed transcripts during the seed development; C – model for transcriptional regulation in the early phase and mid phase of expression. Conserved non-coding sequence regions (CRE1, CRE2 and CRE3) are labelled with open rectangles. Developmental stages are labelled with DPA.

Results presented in this paper indicate that the expression of LMW glutenin genes follow two different patterns. Most of the s- and i-type genes show a continuously increasing expression pattern. The m-type genes, however, demonstrate normal distribution in their expression profiles. We propose that differences found in their promoter sequences should be related to the differences observed in their expression (Figure 6A and 6B). One of the most significant differences is the presence of the complete long endosperm box in most of the gene types showing growing expression, while the rest of the genes carry only a deficient LEB in their promoters. As the deficiency found was mainly due to the absence of one or both of the GCN4-like motifs, it might be possible that in the s- and i- types genes, the CRE2 region ensures tissue-specific expression without reduction in gene expression upon the demanded amino acid levels reached. There was another important difference to observe, namely the presence of an additional SPA bZIP binding site around at −650 nucleotides (in CRE3) of the promoter types of genes with continuously growing expression profiles. This motif was fully absent in the other promoter types. The third most characteristic difference was the presence or absence of a CCAAT box in the basal promoter region (CRE1). The CCAAT box is usually located at around −60–80 nucleotides upstream of the transcriptional start site, but was found at the position −10 in the LMW glutenin gene promoters with maximal expression profiles in the mid-phase of seed filling. Whether CCAAT box has any direct effect on this expression pattern is not known, however, as the CCAAT box does not belong strictly to the basal promoter, it might function as a repressor during the late phases of development.

Based on the possible function of the different *cis*-acting elements and the potential TF interactions the following model can be described (Figure 6C). At the beginning of transcription, elements located in the first conserved non-coding region (CRE1) might fulfil a basal promoter activity initiating the transcription. At around 10–13 days after anthesis MYB and DOF transcription factors might already be expressed and bound to their recognition sites in LMW glutenin genes (CRE1 and CRE2). The functional MYB-DOF TF complexes ensure a moderate level of transcription. This means that at the earlier phases of seed filling, while bZIP transcription factors are not expressed yet to trigger endosperm-specific expression, DOF transcription factors bound to the prolamin boxes might be responsible for trans-activation of the storage protein promoters. This basic level of expression might be moderated with the contribution of MYB transcription factors as suppressors in other vegetative tissues. These fundamental processes might be further fine-tuned by the presence of additional elements, such as CCAAT boxes. As later on, bZIP transcription factors are bound to the GCN4-like motifs, Skn-1 like motifs and SPA bZIP recognition sites, the CRE2 region fulfils its tissue-specific regulator activity. This qualitative function is accompanied with the quantitative moderator effect of interacting bZIP-DOF and bZIP-MYB TF complexes in the CRE3 region and gene expression reaches its maximal level. Based on this model the position of motifs located in the first 1000 nucleotides upstream of the transcriptional start determines their primary role as qualitative or quantitative regulators. Motifs, such as prolamin boxes or GCN4-like motifs are present both in the tissue specific CRE2 motif and the distal CRE3 region. Polymorphisms in the number and combinations of these cis-acting elements can be of significant importance in the diverse levels of production of single LMW glutenin gene types, with the help of further elements in the less conserved promoter regions.

### Conclusions

The study presented here was carried out on a statistically limited sample set, which was due to the limited number of available promoter sequences. Altogether 164 accessions were used in the analyses. Some of the gene types, like MSS1, MEN or IQA were poorly represented. Using this available dataset a model for the transcriptional regulation of LMW glutenin gene expression was developed and confirmed with different statistical approaches. Our results indicate that LMW glutenin gene expression follows two different patterns. A group of gene types, mostly belonging to m-type LMW glutenin genes follow a normal distribution expression pattern, starting to express at around the tenth day after anthesys. They reach their maximum at around 21DPA and the amount of transcripts continuously decreases while reaching the full mature status. The rest of the genes, mainly s- and i-types show a stable level of transcription also after 21DPA. Their promoter profiles and especially the composition of their conserved regulatory element (CRE) regions also highlight some important characteristics, based on which the differences observed in their promoter patterns can be related to their expressional profiles.

Later on, these data can be completed with further sequences when available. However, as this research is an *in silico* analysis the results need to be confirmed with wet-lab experiments. Based on these we suggest to use these results as a guideline, and as a basis for future *in vitro* experiments.

## Supporting Information

File S1 **Fasta files of LMW glutenin gene promoter sequences retrieved from public databases and used for analyses.** Only sequences longer than 30 nucleotides are mentioned.
(TXT)

File S2 **Alignment file of deduced amino acid sequences of the different LMW glutenin gene types.** Gene accessions representing the different gene types are the same as presented in Table 1.
(TIF)

File S3 **Results of two-way joining cluster analysis. X axis represents cis-acting elements, and their position (in brackets) identified in the LMW glutenin gene accessions analysed.** Y axis represents LMW glutenin gene types involved in the analysis. Frequency of cis-acting elements in the individual LMW glutenin gene types are labelled with colours (from 0 – dark green to 1 – dark red)
(TIF)

File S4 **Raw data and G-test results of relationships between LMW glutenin gene types and their expressional profiles carried out based on the methods of Pavlidis and Noble (2003)** [**36**] **.**
(XLS)

File S5 **Raw data and G test results to describe relationships between promoter types and expressional profiles**
(XLS)

File S6 **Motif composition of CRE1, CRE2 and CRE3 regions, expressional data related to CRE groups and G test result of CRE analysis.**
(XLS)
