# Supplementary figures and images for: Role of Conserved Non-Coding Regulatory Elements in LMW Glutenin Gene Expression
Source: PLoS One. 2011 Dec 29;6(12):e29501. doi: 10.1371/journal.pone.0029501 (PMC3248431; doi:10.1371/journal.pone.0029501)

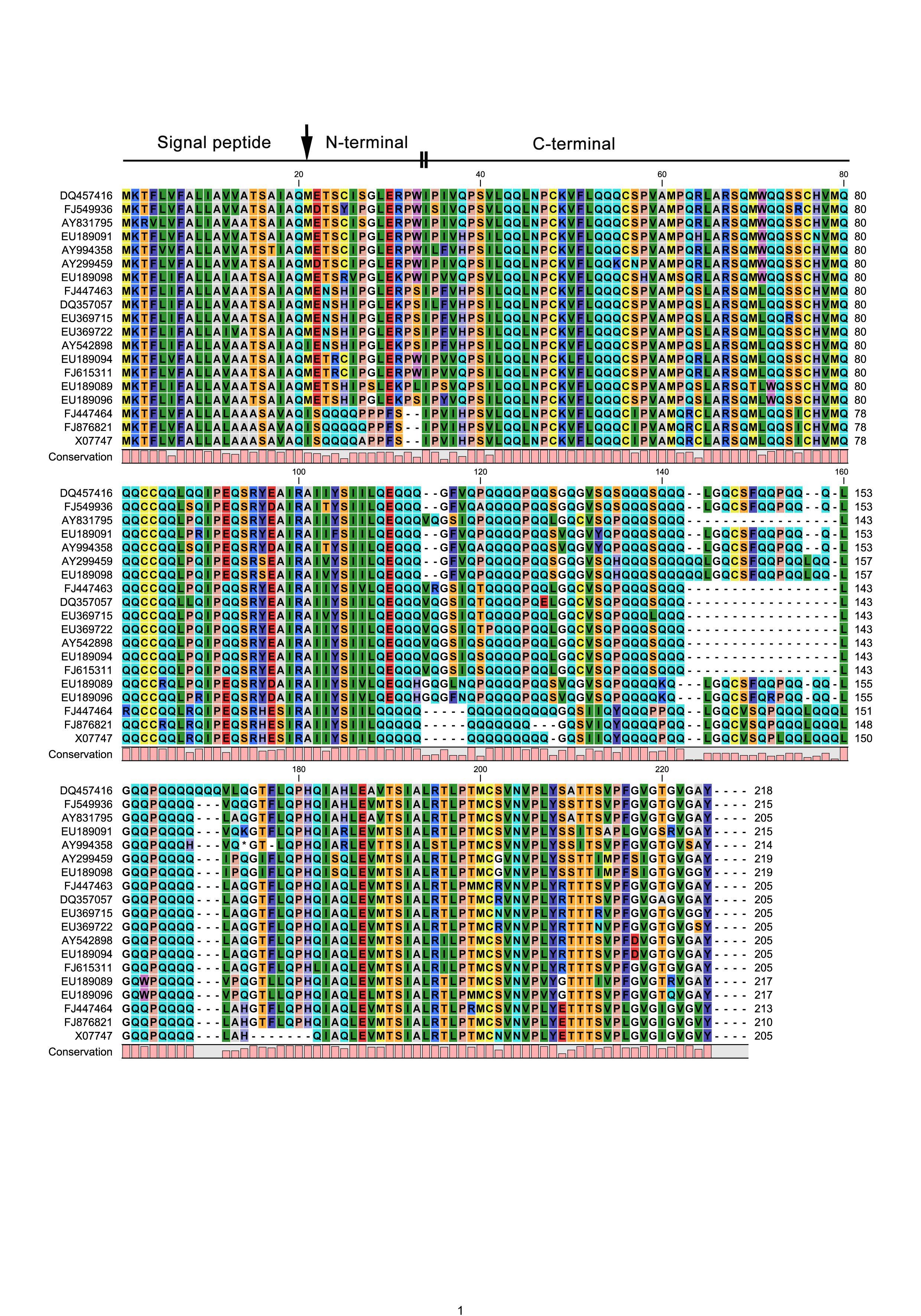

Supplement: File S2 — Alignment file of deduced amino acid sequences of the different LMW glutenin gene types. Gene accessions representing the different gene types are the same as presented in Table 1. (TIF) [file pone.0029501.s002.tif]

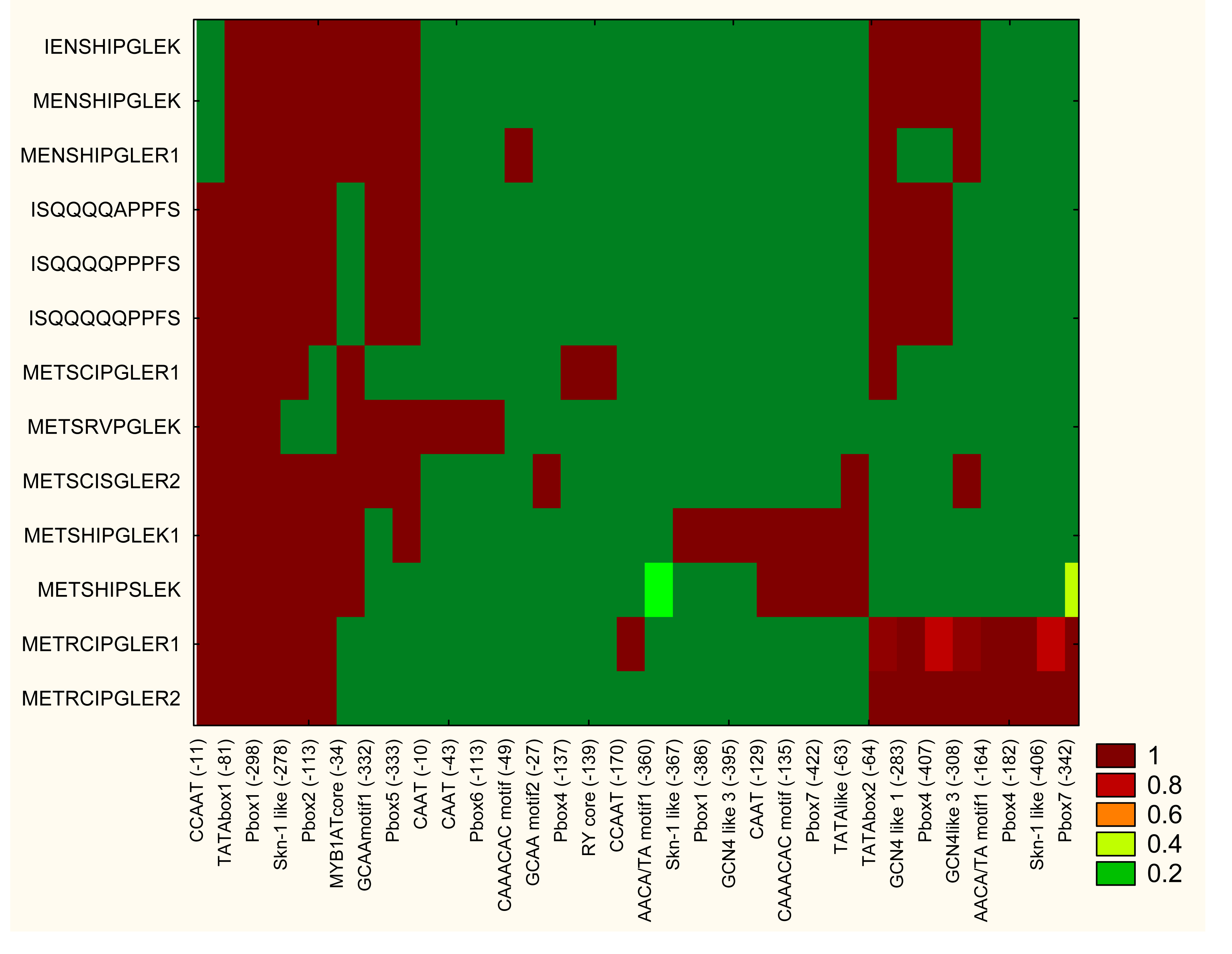

Supplement: File S3 — Results of two-way joining cluster analysis. X axis represents cis-acting elements, and their position (in brackets) identified in the LMW glutenin gene accessions analysed. Y axis represents LMW glutenin gene types involved in the analysis. Frequency of cis-acting elements in the individual LMW glutenin gene types are labelled with colours (from 0 – dark green to 1 – dark red) (TIF) [file pone.0029501.s003.tif]
